# Expression Analysis of Circulating miR-21, miR-34a and miR-122 and Redox Status Markers in Metabolic Dysfunction-Associated Steatotic Liver Disease Patients with and Without Type 2 Diabetes

**DOI:** 10.3390/ijms26062392

**Published:** 2025-03-07

**Authors:** Sanja Erceg, Jelena Munjas, Miron Sopić, Ratko Tomašević, Miloš Mitrović, Jelena Kotur-Stevuljević, Milica Mamić, Sanja Vujčić, Aleksandra Klisic, Ana Ninić

**Affiliations:** 1 Department of Medical Biochemistry, Faculty of Pharmacy, University of Belgrade, 11221 Belgrade, Serbia; sanja.erceg@pharmacy.bg.ac.rs (S.E.); jelenaj@pharmacy.bg.ac.rs (J.M.); miron.sopic@pharmacy.bg.ac.rs (M.S.); jelena.kotur@pharmacy.bg.ac.rs (J.K.-S.); sanjavujcic@icloud.com (S.V.); 2 Faculty of Medicine, University of Belgrade, 11000 Belgrade, Serbia; ratko.tomasevic@med.bg.ac.rs; 3 Department of Gastroenterology and Hepatology, Clinic for Internal Medicine, Clinical Hospital Center Zemun, 11080 Belgrade, Serbia; 4 Clinical Department for Gastroenterology and Hepatology, University Medical Center Zvezdara, 11120 Belgrade, Serbia; dr.milosh.mitrovic@gmail.com; 5 Department of Laboratory Diagnostics, Clinical Hospital Center Zemun, 11080 Belgrade, Serbia; mamicmilics8@gmail.com; 6 Faculty of Medicine, University of Montenegro, 81000 Podgorica, Montenegro; aleksandranklisic@gmail.com; 7 Center for Laboratory Diagnostics, Primary Health Care Center, 81000 Podgorica, Montenegro

**Keywords:** MASLD, circulating miRNA, oxidative stress, redox status, type 2 diabetes

## Abstract

Metabolic dysfunction-associated steatotic liver disease (MASLD), a hepatic form of metabolic syndrome, often co-occurs with type 2 diabetes (T2D) and now affects approximately 30% of the global population. MASLD encompasses conditions from simple steatosis to metabolic dysfunction-associated steatohepatitis, with oxidative stress (OS) driving progression through inflammation. This study analyzes the expression levels of circulating miRNAs and redox status markers in MASLD patients with and without T2D, exploring their potential as disease biomarkers. The expressions of miR-21, miR-34a, and miR-122 were analyzed in the platelet-poor plasma of 147 participants, divided into three groups: MASLD + T2D (48), MASLD (50), and a control group (49). Total oxidant status (TOS), total antioxidant status (TAS), ischemia-modified albumin (IMA), and superoxide anion radical (O_2_^•−^) were measured in serum and plasma. Logistic regression showed that miR-21, miR-34a, TOS, TAS, O_2_^•−^, and IMA were positive predictors of MASLD, while miR-21 and TAS were negative predictors of T2D in MASLD. Although miR-122 did not show a significant association with either condition, in combination with miR-34a and other markers such as lipid status and liver enzymes, a new significant predictor of MASLD was identified. Circulating miRNAs in combination with redox status markers, lipid status and liver enzymes show potential as MASLD biomarkers.

## 1. Introduction

In the last ten years, the prevalence of metabolic dysfunction-associated steatotic liver disease (MASLD) has increased considerably, especially in industrialized countries. According to current estimates, MASLD now affects around 30 percent of the world’s population [1]. In June 2023, the disease formerly known as non-alcoholic fatty liver disease (NAFLD) was renamed MASLD in the Delphi Consensus Statement. According to this consensus, MASLD is diagnosed based on the presence of hepatic steatosis detected by imaging or biopsy, along with at least one of five cardiometabolic risk factors [2]. Despite this revision, much of the existing literature still refers to NAFLD, and both terms may be used interchangeably in the context of past research [3].

As a leading contributor to chronic liver disease, MASLD manifests in two main forms, as follows: simple steatosis, characterized by the presence of fat accumulation in more than 5% of hepatocytes, and metabolic dysfunction-associated steatohepatitis (MASH), formerly known as non-alcoholic steatohepatitis (NASH), an advanced stage associated with inflammation and varying degrees of fibrosis [2,4,5,6]. MASLD is now considered the hepatic form of metabolic syndrome, a group of interrelated risk factors that include central obesity, insulin resistance, dyslipidemia, and hypertension [7]. Type 2 diabetes (T2D), rooted in insulin resistance, often occurs alongside MASLD [8].

Excessive lipid accumulation in hepatocytes induces oxidative stress (OS), defined by an imbalance between the production of reactive oxygen species (ROS) and antioxidant defense mechanisms. OS, along with inflammatory mediators released by adipose tissue and immune cells infiltrating the liver, triggers an inflammatory response leading to MASH. The severity of steatosis correlates with OS and inflammation [9,10].

There is no doubt that the molecular basis of MASLD is intricate, involving numerous signaling molecules participating in hepatic metabolism, oxidative processes, inflammation, and fibrosis [11]. As in other complex diseases, the development and progression of MASLD appears to be influenced by epigenetic mechanisms, including micro ribonucleic acids (miRNAs). These are short, non-coding RNA molecules of approximately 22 nucleotides that regulate gene expression through RNA silencing—the degradation of target messenger RNA or inhibition of translation, but in some cases also through the activation of translation [12,13]. The presence of miRNAs in the bloodstream can result from cell secretion or from cell death processes such as apoptosis, necrosis, tumors or trauma [14]. These circulating miRNAs act as messengers that facilitate paracrine and long-distance cell communication [15,16]. They are remarkably stable, resistant to ribonucleases, temperature and pH fluctuations and repeated freeze–thaw cycles, in part due to their binding to argonaute proteins or high-density lipoprotein (HDL), or their encapsulation in extracellular vesicles (EVs) [17,18,19].

The combination of multiple circulating miRNAs has the potential to improve diagnostic accuracy in the assessment of MASLD. Liver biopsy, the current gold standard for the diagnosis and staging of MASLD, is hampered by its invasive nature and associated complications. Imaging techniques such as ultrasound and computed tomography are limited in utility in detecting mild hepatic steatosis. Therefore, there is a need for the development of reliable non-invasive biomarkers for MASLD diagnosis [20,21]. Importantly, circulating miRNAs can be detected using polymerase chain reaction (PCR), which provides significantly greater sensitivity compared to protein biomarkers [22].

In this study, we focused on miR-21, miR-34a and miR-122, as they are involved in important pathophysiological processes associated with the development and progression of MASLD, including the dysregulation of lipid metabolism, insulin resistance and inflammation. Each of these miRNAs has been shown to play a distinct role in these mechanisms. The upregulation of miR-21 occurs early in the development of hepatic metabolic disorders, and is associated with steatosis and insulin resistance in both obese individuals and rodent models [23]. MiR-21 promotes hepatic steatosis and fibrosis by targeting *peroxisome proliferator-activated receptor alpha* (*PPARα*) and activating hepatic stellate cells (HSCs) [24,25,26]. MiR-34a exerts its effects by downregulating *sirtuin1* (*SIRT1*), which decreases lipid oxidation and increases lipogenesis and inflammation, and contributes to fibrosis via HSC activation [27,28]. The inhibition of miR-34a in animal models significantly reduced hepatic glucose production and gluconeogenic gene expression [29]. Finally, miR-122, the most abundant miRNA in the liver, shows a dynamic expression pattern, increasing in the early stages of MASLD and decreasing as the disease progresses [24,30]. Although the role of miR-122 is controversial, it influences insulin sensitivity and lipid metabolism, with both a protective and a pathogenic role being described in the literature [24].

In our study, we investigated whether there are different circulating miRNA expression profiles in MASLD patients with and without T2D compared to the control group (CG). We also wanted to determine whether these miRNAs either alone or in combination with other markers, such as redox status markers, could be associated with the development of MASLD and T2D in MASLD.

## 2. Results

The general data of the studied populations are shown in Table 1. Patients with MASLD + T2D were significantly older compared to patients with MASLD and CG. There were more males among the patients compared to the CG. In both patient groups, body mass index (BMI), waist circumference and systolic blood pressure were significantly higher compared to CG. As expected, the number of participants taking insulin therapy and oral antidiabetic drugs was higher in the group of patients with MASLD + T2D than in the other two groups. More participants in the CG group were physically active than in the group of patients with MASLD, but fewer participants had hypertension and/or cardiovascular disease (CVD) and received antihypertensive and/or CVD therapy than in the other two patient groups. The numbers of smokers, alcohol consumers and participants receiving antihyperlipidemic therapy were similar in all three groups.

Table 2 shows biochemical and redox status markers in the populations studied. As expected, glucose concentrations and glycosylated hemoglobin (HbA1c) levels were significantly higher in patients with MASLD + T2D than in the other two groups. In addition, glucose levels were significantly higher in patients with MASLD than in CG. Patients with MASLD + T2D had higher triglycerides (TG) and lower HDL-cholesterol levels compared to the other groups, and patients with MASLD had lower HDL-cholesterol levels than CG. Liver enzymes (alanine aminotransferase (ALT) and γ-glutamyl transferase (GGT)) and C-reactive protein (CRP) were elevated in both patient groups compared to CG. 

After adjusting for covariates (age, sex, BMI, physical activity, and antihypertensive and/or CVD therapy), redox status markers, including total antioxidant status (TAS), total oxidant status (TOS) and ischemia-modified albumin (IMA), were elevated in both patient groups compared to CG. TAS and TOS levels were the highest in patients with MASLD. Superoxide anion radical (O_2_^•−^) levels were significantly higher in patients with MASLD compared to both the other patient group and CG. The reason why certain covariates, such as waist circumference and hypertension and/or CVD, were not included in the model was to avoid multicollinearity. The Spearman correlation analysis and the Chi-square test revealed significant associations; waist circumference was strongly correlated with BMI (ρ = 0.764, *p* < 0.001), and the presence of hypertension and/or CVD was strongly associated with antihypertensive and/or CVD therapy (*p* < 0.001).

**Figure 1:**
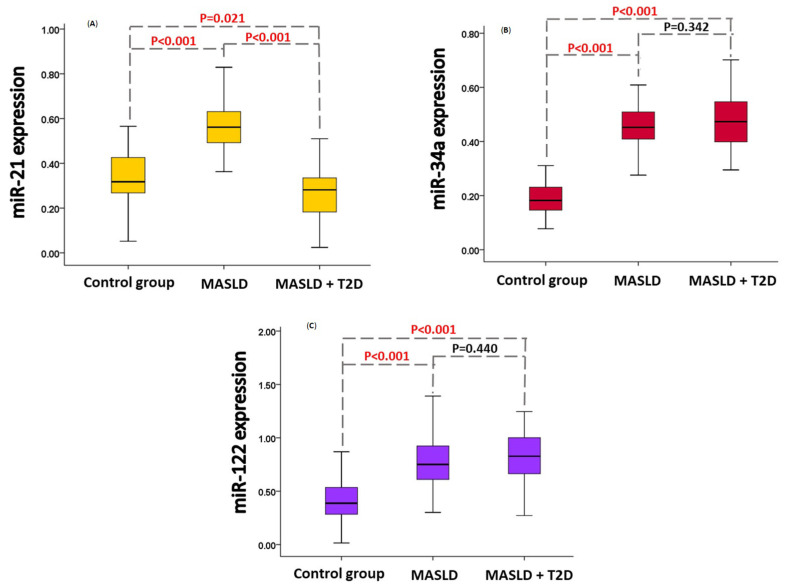
Expressions of miR-21 (**A**), miR-34a (**B**) and miR-122 (**C**) in the studied population. miRNA expression levels are presented as relative expression values calculated using the 2^−ΔCt^ method, where ΔCt is determined as the difference between the Ct value of the miRNA of interest and the Ct value of the exogenous control for each sample. Expression levels of all three miRNAs are adjusted for age, sex, BMI, physical activity, antihypertensives and/or CVD therapy. Statistical comparisons between groups are indicated by *p*-values, with significant differences (*p* < 0.05) highlighted in red.

Similar to redox status markers, miRNA expression levels were adjusted for age, sex, BMI, physical activity, and antihypertensive and/or CVD therapy. The expression of miR-21 was lowest in patients with MASLD + T2D (*p* < 0.001 vs. patients with MASLD; *p* < 0.021 vs. CG) but highest in patients with MASLD (*p* < 0.001 vs. CG) (Figure 1A). The expression of miR-34a was significantly higher in both patient groups compared to CG (*p* < 0.001 in both cases) (Figure 1B). Likewise, miR-122 expression was significantly higher in both patient groups compared to CG (*p* < 0.001 in both cases) (Figure 1C).

In addition to adjusting for potential confounders, we also examined potential associations between miRNA expression and parameters that differed between groups (age, sex, BMI, waist circumference, antihypertensive and/or CVD therapy, and physical activity) using Spearman correlation and univariate binary logistic regression analyses. MiR-34 expression significantly correlated with BMI (*p* = 0.002) and waist circumference (*p* = 0.014), while miR-122 expression showed a significant correlation with BMI (*p* = 0.005). Additionally, miR-21 expression was significantly associated with antihypertensive and/or cardiovascular therapy (*p* = 0.016).

All continuous markers from Table 1 and Table 2 were tested using univariate binary logistic regression analysis to assess the association with MASLD (Table 3). Two groups were included in the analysis—patients with MASLD and those with CG. Increasing the expression levels of miR-21 and -34a by one expression unit increased the risk of developing MASLD by more than 8- and 7-fold, respectively. Nagelkerke R^2^ showed that the expression levels of miR-21 and -34a can explain 12.6% and 14.9% of the variation in the incidence of MASLD, respectively.

Some markers that showed a significant odds ratio (OR) in univariate binary regression analysis (Table 3) were tested as covariates in the multivariate analysis. The first multivariate analysis included BMI, hypertension and CVD presence, glucose, TG, HDL-cholesterol, ALT, GGT, CRP, TAS, TOS, IMA and miR-21 as covariates, while the second multivariate analysis included miR-34a instead of mir-21. Neither miR-21 nor miR-34a showed a significant independent association with MASLD (OR = 0.658 (0.035–12.559); *p* = 0.781 and OR = 0.381 (0.015–9.950); *p* = 0.562). Complete statistical details, including OR, 95% confidence interval (CI), and Nagelkerke R^2^, for all covariates are available in Supplementary Table S1.

Second, we investigated whether the markers were associated with T2D in MASLD by applying a univariate binary logistic regression analysis to both patient groups (Table 4). A one-unit reduction in miR-21 expression level increased the risk of T2D in MASLD by 96.4%. Nagelkerke R^2^ showed that miR-21 expression could explain the 21.8% variation (reduction) in T2D risk in patients with MASLD.

The third multivariate analysis included age, TG, HDL-cholesterol, TAS and miR-21 expression as covariates (Supplementary Table S2). The expression of miR-21 showed a significant independent negative association with T2D in MASLD (OR = 0.050 (0.004–0.668); *p* = 0.023).

**Figure 2:**
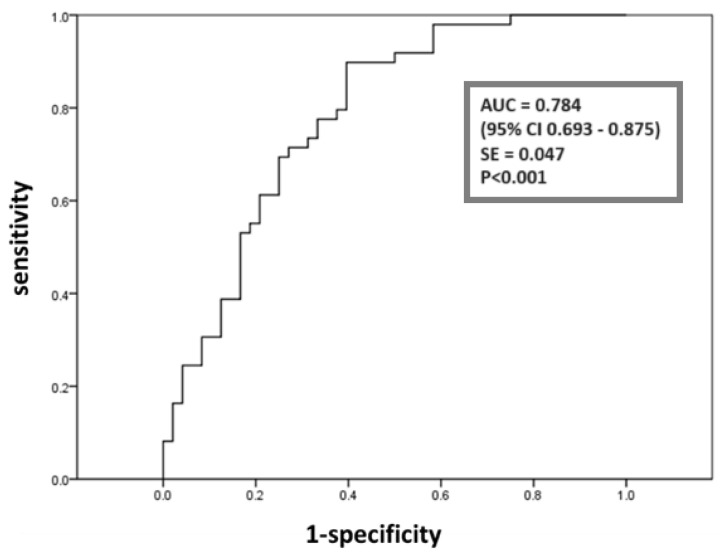
ROC analysis of miR-21 discriminatory power for T2D in MASLD. Abbreviations: AUC, area under the curve; CI, confidence interval; SE, standard error.

Although miR-21 was independently associated with T2D, we aimed to further evaluate its ability to discriminate patients with steatosis who already had T2D using receiver operating characteristic (ROC) analysis. The analysis yielded an area under the curve (AUC) of 0.784, indicating acceptable accuracy according to Hosmer and Lemeshow [31] (Figure 2). Furthermore, we assessed the ability of the markers from the third multivariate analysis (age, TG, HDL-cholesterol, TAS and miR-21) to discriminate MASLD patients with T2D, yielding an AUC of 0.831 (0.738–0.924), which reflects excellent accuracy based on the same criteria [31].

In the principal component analysis (PCA) the Kaiser–Meyer–Olkin (KMO) measure of adequacy of the sample was 0.753 and the Bartlett’s test for sphericity was significant (*p* < 0.001). This analysis identified four significant factors, collectively explaining 58% of the variation in the investigated markers (Table 5). The epigenetic liver-specific-related factor explained 23.3% of variance, and it was associated with positive loadings of miR-122, miR-34a, ALT, GGT and TG levels. The cardiometabolic antioxidant-related factor explained 14.4% variance, and it was associated with positive loadings of TAS, BMI and CRP and negative loadings of HDL-cholesterol. The third factor, redox-related factor, explained 10.2% of variance, and it was related to positive loadings of TOS and IMA and negative loadings of O_2_^•−^. The fourth factor, age–epigenetic-related factor, explained 10.1% of variance, and it was related to positive loadings of age and negative loadings of miR-21.

Furthermore, we used factor scores in univariate binary logistic regression analysis to assess their predictive abilities for MASLD and T2D in MASLD (Table 6). The epigenetic liver-specific-related factor was positively associated with MASLD (OR = 2.915, *p* = 0.026). Increased epigenetic liver-specific-related factor values were associated with an almost 3 times greater probability of MASLD. This factor explained the variation in MASLD of 16.5%, as demonstrated by the Nagelkerke R^2^ of 0.165. Also, the cardiometabolic antioxidant-related factor was positively associated with MASLD (OR = 4.604, *p* < 0.001). Increased cardiometabolic antioxidant-related factor values were associated with more than 4 times greater probability of MASLD. This factor explained the variation in MASLD of 34.9%, which was demonstrated by the Nagelkerke R^2^ = 0.349. The redox-related factor and age–epigenetic-related factor were not associated with MASLD. On the contrary, only the age–epigenetic-related factor was positively associated with T2D in MASLD (OR = 3.280, *p* = 0.002). Increased age–epigenetic-related factor values were associated with more than 3 times greater probability of T2D in MASLD patients. This factor explained the variation in T2D in MASLD of 27.7%, which was demonstrated by the Nagelkerke R^2^ of 0.277.

To evaluate and compare the predictive abilities of PCA-derived factors with those of traditional hepatic steatosis indices for MASLD, we also performed univariate binary logistic regression for the hepatic steatosis index (HSI) and fatty liver index (FLI). Both HSI and FLI were positively associated with MASLD (OR = 1.288, *p* < 0.001; OR = 1.052, *p* < 0.001, respectively). Nagelkerke R^2^ showed that HSI and FLI explained 37.6% and 39.4% of the variation in the incidence of MASLD, respectively. Detailed results are presented in Supplementary Table S3.

## 3. Discussion

Circulating miRNAs represent promising biomarkers due to their wide distribution, with specific miRNAs reflecting different pathophysiological conditions, including MASLD and T2D [20]. Since a considerable number of patients had both MASLD and T2D, which is consistent with global epidemiological data [32], we formed three study groups: patients with MASLD, patients with MASLD and T2D, and a CG. The expression of miR-21 was lowest in patients with MASLD + T2D, but highest in patients with MASLD (Figure 1A). The expressions of miR-34a and miR-122 were significantly higher in both patient groups compared to the CG (Figure 1B,C). We explored direct associations between miRNA expression and parameters that differed between groups, which also represent potential confounders. MiR-34 expression significantly correlated with BMI (*p* = 0.002) and waist circumference (*p* = 0.014), while miR-122 expression showed a significant correlation with BMI (*p* = 0.005). Additionally, miR-21 expression was significantly associated with antihypertensive and/or cardiovascular therapy (*p* = 0.016). To assess whether these and other factors influenced miRNA expression differences across groups, all three miRNAs were adjusted for age, sex, BMI, physical activity, and antihypertensive and/or CVD therapy. Waist circumference was excluded from the adjustment model due to its high correlation with BMI to avoid multicollinearity problems. The same applies to hypertension and/or CVD, and antihypertensive and/or CVD therapy. This approach ensured that the observed differences in miRNA expression remained independent of potential confounders, providing a more robust interpretation of the results.

The study by Yamada et al. [9] showed higher miR-21 levels in the serum of MASLD patients compared to CG, which is consistent with our findings (Figure 1A). The hepatic expression of miR-21 has been shown to increase with the progression of MASLD [24]. Our results indicate that patients with MASLD had higher levels of OS and inflammatory markers compared to the CG, including TOS, IMA, O_2_^•−^, and CRP (Table 2), suggesting a more advanced disease state. These results will be further discussed. The role of miR-21 in disease progression is supported by the fact that miR-21 promotes fibrosis through the activation of HSCs and the conversion of hepatocytes into myofibroblasts [26,33,34,35]. In addition, the long-term inhibition of miR-21 has been shown to reduce obesity in animal models, highlighting the potential use of miR-21 as a therapeutic target to reduce MASLD activity [36]. We found only one study with an MASLD + T2D patient group that showed an upregulation of miR-21 in plasma compared to CG, which contradicts our results (Figure 1A) [37]. Although miR-21 promotes insulin resistance [38], it is important to consider that our patients with T2D mostly receive metformin therapy, which has been shown to downregulate miR-21 in the circulation [39]. This could be the reason for the lowest miR-21 expression in our patients with MASLD + T2D (Figure 1A). Univariate binary regression analysis showed that miR-21 is a significant predictor of the development of both MASLD and T2D in MASLD (Table 3 and Table 4). Using multivariate logistic regression analysis, we identified miR-21 as the only significant negative independent predictor of T2D in MASLD (Table S2). We further investigated the ability of miR-21 to discriminate between MASLD patients with and without T2D. ROC analysis showed that miR-21 effectively discriminated MASLD + T2D patients from MASLD patients, achieving an AUC of 0.784 (0.693–0.875), indicating acceptable accuracy [31] (Figure 2). When miR-21 was combined with other significant markers from the third multivariate analysis (age, TG, HDL cholesterol and TAS), the AUC increased to 0.831 (0.738–0.924), indicating excellent accuracy by the same criteria [31]. Although oral glucose tolerance test, fasting plasma glucose, and HbA1c remain the traditional methods for diagnosing T2D [40], our study demonstrated that miR-21, in combination with other markers, could serve as a complementary biomarker, providing additional insights into the metabolic and inflammatory disturbances contributing to MASLD progression. To date, no study has investigated the impacts of these epigenetic factors in combination with other markers for predicting the development of T2D in MASLD.

Previous studies have reported an increase in circulating miR-34a levels in MASLD [9,41,42] and in T2D with hepatic fatty infiltration compared to a CG [37], which is consistent with our findings (Figure 1B). By downregulating *SIRT1*, an NAD^+^-dependent deacetylase, miR-34a, increases the acetylation of multiple target genes, leading to decreased lipid oxidation while promoting lipogenesis and inflammation [27]. Similar to miR-21, miR-34a increases with disease progression, which is not surprising as miR-34a promotes HSC activation and fibrosis [24,28,43]. In addition, miR-34a induces hepatic glucose production and gluconeogenic gene expression, contributing to insulin resistance [29]. The univariate binary logistic regression analysis showed that miR-34a is a significant positive predictor for the development of MASLD (Table 3).

Our study has demonstrated a significant upregulation of miR-122 in patients with MASLD + T2D compared to the CG (Figure 1C), consistent with the findings of Ye et al. [44]. Similarly, the Bruneck study [45], the first population-based research on miR-122 as a biomarker, identified a strong association between miR-122 and the onset of metabolic syndrome and T2D, even after adjusting for various lifestyle and demographic factors. Furthermore, in our analysis, after adjusting for covariates, miR-122 became upregulated in patients with MASLD compared to the CG (Figure 1C), aligning with findings from Pirola et al. [46], Tobaruela-Resola et al. [47], and Yamada et al. [9]. We must highlight the ongoing inconsistencies in the literature regarding the precise role of miR-122 in MASLD. By targeting *SIRT1*, miR-122 stimulates lipogenesis; however, by targeting TG biosynthesis enzymes, it suppresses lipogenesis [48]. *SIRT1* is also a target of miR-34a, yet no evidence of a potential synergistic effect between these two miRNAs on *SIRT1* downregulation has been reported. Compared to miR-21 and miR-34a, miR-122 tends to increase in the early stages of NAFLD, but declines as the disease progresses to NASH and cirrhosis [24]. However, despite its increased levels in MASLD and MASLD + T2D, univariate binary logistic regression analysis showed no significant association of miR-122 with MASLD or T2D in MASLD (Table 3 and Table 4). Interestingly, after applying PCA, miR-122, together with miR-34a, TG, ALT, and GGT, emerged as an integral component of the epigenetic liver-specific-related factor (Table 5), which will be further discussed.

Considering the multifactorial nature of MASLD and the critical role of OS in its progression, we analyzed markers of redox status in the studied groups. All of them were adjusted for age, sex, BMI, physical activity and antihypertensive and/or CVD therapy. TOS was significantly higher in both patient groups compared to CG (Table 2), supporting the idea that MASLD and especially MASLD + T2D exacerbate OS and vise versa. This state of increased OS necessitates a stronger antioxidant defense response, both enzymatic and non-enzymatic, to neutralize OS and minimize cellular damage [49,50]. In our study, TAS was significantly higher in both patient groups compared to CG, which probably indicates that the adaptive response was activated (Table 2). We must consider that TAS represents all reducing substances in the blood, including uric acid, bilirubin and total protein [51]. Uric acid and total protein were significantly higher in our patients compared to the CG (Table 2). Therefore, uric acid and total protein likely contributed, at least in part, to the elevated TAS observed in our patients. O_2_^•−^, a primary oxygen radical formed when an oxygen molecule acquires an electron and triggers a cascade of ROS, was higher in patients with MASLD compared to patients with MASLD + T2D and the CG. This aligns with TOS being highest in patients with MASLD, as O_2_^•−^ is a component of it [49,50]. However, no difference in O_2_^•−^ levels was observed between patients with MASLD + T2D and the CG (Table 2). One of the explanations could be the high reactivity and short half-life of ROS, which make them difficult to detect in the bloodstream. Alternatively, ROS can be measured by assessing the products resulting from the damage they cause to different biomolecules [52]. For example, IMA resulting from conformational changes at the N-terminal end of albumin due to OS was significantly higher in both patient groups compared to CG (Table 2) [53]. This finding is consistent with those from previous studies showing increased IMA levels in patients with chronic liver disease and T2D compared to CG [53,54]. Univariate binary logistic regression showed that TOS, TAS, O_2_^•−^ and IMA were all positive predictors of MASLD, with TAS being the only negative predictor of T2D in MASLD (Table 3 and Table 4).

Our main goal was to investigate whether circulating miR-21, miR-34a, and miR-122, along with inflammatory markers, redox status markers, lipid profile, and liver enzymes, were linked to the presence of MASLD and T2D in patients with MASLD. We employed PCA to create combinations of factors that could be analyzed for their association with the development of MASLD and T2D in MASLD using univariate binary logistic regression. This approach has not been applied in previous studies for this purpose. Two such combinations that were associated with the development of MASLD were epigenetic liver-specific-related factor and cardiometabolic antioxidant factor. The epigenetic liver-specific-related factor comprised miR-122, miR-34a, ALT, GGT and TG (Table 5). Although miR-122 alone was not a significant predictor for the development of MASLD (Table 3), it was found to be when in combination with miR-34a, ALT, GGT and TG (Table 6). The liver-specific-related aspect of the epigenetic liver-specific-related factor refers to the liver enzymes ALT and GGT, as well as TG. Elevated levels of TG were observed in patients with both MASLD and T2D compared to those with MASLD alone and the CG. ALT and GGT were slightly increased in both patient groups compared to CG, although the values for all participants were within the reference intervals (Table 2). Normal levels of liver enzymes were observed in patients across the spectrum of MASLD, limiting their utility in the prediction of MASLD [55]. However, in combination with these markers, they could become a part of a diagnostic panel. The cardiometabolic antioxidant factor included TAS, BMI, CRP and HDL-cholesterol (Table 5). Only HDL-cholesterol showed a negative loading in the cardiometabolic-antioxidant factor, which is consistent with the fact that individuals with MASLD often have low HDL-cholesterol levels, which are associated with an increased risk of developing T2D [56]. BMI and CRP are also part of this factor, as obesity is one of the main risk factors for the development of insulin resistance and MASLD, while CRP is an indicator of inflammation that occurs in MASH [6,7]. Both groups of our patients had significantly higher BMI compared to CG (Table 1 and Table 2). TAS was included in the cardiometabolic antioxidant factor with positive loading, probably due to the aforementioned compensatory response. Only the age–epigenetic-related factor involving the downregulation of miR-21 expression and age was found to be a significant predictor for the development of T2D in MASLD. This means that miR-21 is an independent predictor of T2D in MASLD, as well as a factor further confirming the implication of miR-21 in the pathogenesis of T2D in MASLD.

We also calculated traditional indices of hepatic steatosis, HSI [57] and FLI [58], to compare their predictive ability for MASLD with the predictive ability of the epigenetic liver-specific-related factor and the cardiometabolic antioxidant-related factor. HSI was calculated using the ALT/AST ratio, BMI, and additional points for female sex and T2D status [57]. FLI was calculated using TG, BMI, GGT, and waist circumference [58]. The influences of all these factors have been examined individually and tested in clinical practice, and thus have a recognized advantage. However, no study has yet integrated epigenetic markers with well-established markers to develop novel predictive factors. Even though traditional indices (HSI and FLI) have a higher Nagelkerke R^2^, our factors exhibit higher OR values, indicating a stronger individual association with MASLD. While Nagelkerke R^2^ reflects the overall predictive power of the model, OR measures the strength of association between a specific factor and MASLD. This suggests that our factors may have a stronger individual impact. Moreover, the cardiometabolic antioxidant-related factor explains disease variance similarly to HSI and FLI (0.349, 0.376, and 0.394, respectively) (Table S3).

This case–control study has several limitations. The sample was small and did not include participants who had undergone liver biopsy, resulting in insufficient data on the severity of MASLD. In addition, there was a lack of precise staging of the disease due to the absence of more sophisticated imaging techniques. Another important limitation is the potential influence of confounding factors such as age, sex, BMI, lifestyle factors and pharmacological treatments on miRNA expression and redox status markers. Although we adjusted for these variables in our analysis, their impact on miRNA expression and redox status markers cannot be entirely excluded. Future studies with larger cohorts, stratified analyses, and follow-up periods are needed to further validate and extend these findings.

## 4. Materials and Methods

### 4.1. Patients

The study included 147 participants who underwent ultrasound examination at the Clinical Hospital Center “Zemun” and University Medical Center “Zvezdara” from January 2020 to March 2023. The subjects were divided into three groups: 48 patients with MASLD and T2D (MASLD + T2D), 50 patients with MASLD and 49 apparently healthy controls (CG). T2D was diagnosed according to American Diabetes Association Criteria [40]. All participants gave written informed consent prior to participation. The entire study was designed and conducted in accordance with the principles of the Declaration of Helsinki [59]. The study protocol was approved by the Ethics Committees of the University of Belgrade—Faculty of Pharmacy, the Clinical Hospital Center “Zemun” and the University Medical Center “Zvezdara” (approval numbers: 835/2, 733/1 and 3/2022/1 of 11 April 2022, 17 October 2019 and 18 March 2022, respectively).

During the examination, the subjects filled out a questionnaire containing demographic and clinical data (age, gender, body weight, body height, waist circumference, systolic and diastolic blood pressure, presence of other diseases, diseases of relatives, medication taken) as well as data on lifestyle habits, including smoking status, alcohol consumption and physical activity. BMI was calculated from body weight (kg)/(height (m))^2^.

The exclusion criteria from the study were as follows: all types of viral hepatitis, autoimmune hepatitis, alcoholic liver disease, primary biliary cirrhosis, sclerosing cholangitis, type I diabetes, Wilson’s disease, α1-antitrypsin deficiency, decompensated cirrhosis, current or previous alcohol abuse, liver transplantation, hepatocellular cancer (HCC) and other malignancies, and renal disease.

### 4.2. Blood Biochemistry

Fasting serum and whole blood containing the anticoagulant K_2_EDTA were taken from all participants at the Clinical Hospital Center “Zemun” and the University Medical Center “Zvezdara”. Serum and plasma were separated from blood cells within one hour by centrifugation (1500 rcf, 10 min). Markers of lipid status (total cholesterol, TG, HDL-cholesterol), glucose, urea, creatinine, uric acid, total protein, albumin, direct and total bilirubin were measured in serum using routine spectrophotometric methods on DxC 700 AU and DxC 480 AU automated analyzers (Beckman Coulter, Brea, CA, USA). CRP, ALT and GGT were also assessed in serum, with CRP determined by the immunoturbidimetric method and ALT and GGT by enzymatic methods on the same analyzers. Low-density lipoprotein (LDL)-cholesterol was calculated using the Friedewald formula [60]. Whole blood samples were analyzed for HbA1c using the immunoturbidimetric method. Vacutainers not used for initial analyses, one serum and one whole blood per participant, were transported to the University of Belgrade—Faculty of Pharmacy, where they were centrifuged under the same conditions (1500 rcf, 10 min). Aliquots of serum and plasma were then frozen at −80 °C until further analyses could be conducted.

The traditional indices of hepatic steatosis were calculated using following formulae:

- HSI = 8 × (ALT/AST ratio) + BMI (+2, if female; +2, if T2D) [57]
- FLI = (e^0.953 × ln(TG) + 0.139 × BMI+0.718 × ln(GGT)+0.053 × waist circumference − 15.745)^/(1 + e^0.953 × ln(TG)+0.139 × BMI + 0.718 × ln(GGT) + 0.053 × waist circumference − 15.745^) × 100 [58]

### 4.3. Redox Status Markers

TAS, TOS and IMA were determined from the serum spectrophotometrically. In the TAS test, 2,2′-azino-bis(3-ethylbenzothiazoline-6-sulfonic acid) (ABTS) is used as a chromogen, which, in combination with peroxidase and H_2_O_2_, forms a stable blue-green ABTS^•+^ radical cation that is measured at 600 nm. The antioxidants present in the sample suppressed this color formation in proportion to their concentrations [61]. TOS was assessed by the o-dianisidine method [62]. The IMA assay developed by Bar-Or et al. [63] is based on the measurement of unbound cobalt after incubation with the patient’s serum, and its values are expressed as absorbance units (ABSU). On the other hand, O_2_^•−^ was determined from the plasma following the procedure outlined by Auclair and Voisin. This involved assessing the rate of nitroblue tetrazolium (NBT) reduction to determine the rate of O_2_^•−^ generation [64]. The ILAB 650 analyzer (Instrumentation Laboratory, Milan, Italy) was used for the determination of TAS, TOS and O_2_^•−^, and the SPECTROstar Nano (BMG Labtech, Ortenberg, Germany) was used for IMA.

### 4.4. miRNA Isolation

Here, 250 μL of plasma was centrifuged a second time in a fixed-angle rotor (10,000 rcf, 10 min, 4 °C) to obtain platelet-poor plasma (PPP), which served as a sample for total RNA isolation. Then, 200 μL of the supernatant was carefully transferred to a new microtube without disturbing the precipitate. In this way, we could ensure that residual cell debris or particles that might still be present in the plasma were removed, and we obtained a cell-free plasma sample, which increased the purity of miRNA isolation. The samples were stored at −80 °C until further use.

Total RNA was isolated using the miRNeasy Serum/Plasma Kit (Qiagen, Hilden, Germany). 5′-phosphorylated synthetic RNA oligo C. Elegans miRNA, cel-miR-39-3p (Microsynth, Balgach, Switzerland), was used as a spike-in or exogenous control to match RNA extraction efficiency. Then, 3.5 μL of this spike-in control (1.6 × 108 copies/μL working solution) was first added to 1 mL of QIAzole lysis reagent, after which this mixture was added to each sample, and isolation was continued according to the manufacturer’s protocol. Total RNA was eluted from the column with 14 μL of water to obtain approximately 12 μL of eluate. After isolation, the samples were stored at −80 °C until further use.

### 4.5. miRNA Quantification

Reverse transcription (RT) and real-time PCR (qPCR) were performed using the 7500 Real-Time PCR System (Thermo Fischer Scientific, Waltham, MA, USA). cDNA synthesis and qPCR reactions were performed using the TaqMan Advanced miRNA cDNA Synthesis Kit (Thermo Fisher Scientific, Waltham, MA, USA), HOT FIREPol^®^ Probe qPCR Mix Plus (Solis Biodyne, Tartu, Estonia) and TaqMan Advanced miRNA Assays: 477975_mir for miR-21-5p, 478048_mir for miR-34a-5p, and 477855_mir for miR-122-5p (Thermo Fisher Scientific, Waltham, MA, USA) according to the manufacturer’s protocol. cDNA synthesis included a poly(A) tailing reaction (37 °C for 45 min, 65 °C for 10 min, pause at 4 °C), an adaptor ligation reaction (16 °C for 60 min, pause at 4 °C), a reverse transcription (RT) reaction (42 °C for 15 min, 85 °C for 5 min, pause at 4 °C), and miRNA amplification (95 °C for 5 min, 14 cycles at two temperatures—95 °C for 3 s and 60 °C for 30 s, 99 °C for 10 min, hold at 4 °C). According to the manufacturer’s instructions, the cDNA template was diluted 1:10 in 0.1X TE buffer. The dilution was used for quantitative PCR reactions that included enzyme activation (95 °C for 15 s), 40 cycles of denaturation (95 °C for 15 s), and annealing (60 °C for 1 min). All samples were run in triplicate. Normalization was performed using cel-miR-39-3p as the exogenous control. Threshold cycle values (Ct) were used for normalization and Ct difference values (dCt) were calculated.

### 4.6. Statistical Analysis

Statistical analyses were performed with SPSS version 29 (SPSS Inc, Chicago, USA). The distribution of continuous markers was tested using the Shapiro–Wilk test, and the differences between markers were assessed using one-way analysis of variance with the Tukey–Kramer post-hoc test for normally distributed data and the Kruskal–Wallis test with a Mann–Whitney U post-hoc test for non-normally distributed data. Normally distributed data were expressed as mean ± standard deviation (SD) and non-normally distributed data as median (interquartile range). Categorical data were tested using the Chi-square test for contingency tables and expressed as absolute and relative frequencies. 

Spearman correlation analysis and univariate binary logistic regression analysis were used to examine potential associations between miRNA expression and parameters that differed between groups (age, sex, BMI, waist circumference, antihypertensive and/or CVD therapy and physical activity). In addition, miRNAs and redox status markers were adjusted for age, sex, BMI, antihypertensive and/or CVD therapy and physical activity using predictive values from a regression model to assess whether these factors influenced differences in miRNA expression between groups. 

Univariate and multivariate binary logistic regression analyses were used to test whether there were significant associations between continuous markers and MASLD (categorical dichotomous variables: 0—no MASLD and 1—MASLD) and continuous markers and T2D in MASLD (categorical dichotomous variables: 1—MASLD and 2—MASLD + T2D). Data are presented as odds ratios (OR) and 95% CI. The explained variations in dependent variables are represented by Nagelkerke R^2^ values. 

PCA with varimax rotation was used to reduce the number of variables to an appropriate number of factors corresponding to the level of variation. Factor extraction was determined when the eigenvalue was greater than 1. The criterion for inclusion of the variables in each factor was factor loadings greater than 0.5. PCA analysis also allowed us to calculate scores for the factors and to use these scores in the subsequent binary logistic regression analysis to test their associations with MASLD and T2D in MASLD.

ROC curve analysis was used to identify clinical markers that may reveal T2D in MASLD. Data are presented as AUC, 95% CI and standard error (SE).

The differences in marker values and their correlations were considered statistically significant at a significance level (*p*) of less than 0.05.

## 5. Conclusions

Circulating miR-21 was lowest in patients with MASLD + T2D, but highest in patients with MASLD. Circulating miR-34a and miR-122 were higher in both patient groups compared to CG. Redox status markers (TAS, TOS, IMA) were elevated in both patient groups compared to CG, with TAS and TOS being highest in patients with MASLD. O_2_^•−^ was higher in patients with MASLD compared to patients with MASLD + T2D and CG. Mir-21 and miR-34a, as well as redox status markers including TAS, TOS, O_2_^•−^ and IMA, showed a positive association with the development of MASLD, while miR-21 and TAS showed a negative association with the development of T2D in MASLD. MiR-21, in combination with age, TG, HDL-cholesterol and TAS, successfully discriminated between MASLD patients with and without T2D by achieving an AUC of 0.831, indicating excellent accuracy. We identified two factors as positive predictors of MASLD, namely, epigenetic liver-specific-related factor (miR-122, miR-34a, ALT, GGT and TG) and cardiometabolic antioxidant factor (TAS, BMI, CRP and HDL-cholesterol). Larger cohorts are required to validate these findings.

## Figures and Tables

**Table 1:** Demographic and clinical characteristics of the population studied.

Marker	CG N = 49	MASLD N = 50	MASLD + T2D N = 48	*p*
Age, years	50 ± 14	50 ± 13	58 ± 13 a†, b†	0.006
Sex (males), N (%) **	13 (26.5)	28 (56.0) a#	22 (45.8) a†	0.011
BMI, kg/m^2^ *	25.1 (22.2–27.0)	29.1 (26.4–31.3) a#	30.4 (26.8–31.4) a#	<0.001
Waist circumference, cm	84.7 ± 10.1	96.9 ± 12.7 a#	99.6 ± 14.4 a#	<0.001
Systolic blood pressure, mmHg *	120 (115–130)	130 (120–140) a‡	130 (120–140) a†	0.016
Diastolic blood pressure, mmHg *	80 (70–83)	80 (70–90)	80 (70–86)	0.153
Smoking status (yes), N (%) **	13 (26.5%)	20 (40.0%)	22 (45.8%)	0.130
Alcohol consumption (yes), N (%) **	18 (36.7%)	9 (18.0%)	11 (22.9%)	0.088
Physical activity (yes), N (%) **	27 (55.1%)	14 (28.0%) a#	17 (35.4%)	0.017
Hypertension and/or CVD (yes), N (%) **	11 (22.4%)	31 (62.0%) a#	25 (52.1%) a‡	<0.001
Insulin therapy (yes), N (%) **	0 (0.0%)	0 (0.0%)	19 (39.6%) a#, b#	<0.001
Oral antidiabetic therapy (yes), N (%) **	0 (0.0%)	0 (0.0%)	36 (75.0%) a#, b#	<0.001
Antihyperlipidemic therapy (yes), N (%) **	2 (4.1%)	5 (10.0%)	7 (14.6%)	0.210
Antihypertensives and/or CVD therapy (yes), N (%) **	10 (20.4%)	30 (60.0%) a#	21 (43.8%) a†	<0.001

Abbreviations: CG, control group; T2D, type 2 diabetes; BMI, body mass index; CVD, cardiovascular disease. Data are presented as Arithmetic mean ± SD; *p*-value for ANOVA; * median (interquartile range) and *p*-value for the Kruskal–Wallis test; a—significant difference between MASLD and MASLD + T2D compared to the CG; b—significant difference between MASLD + T2D and MASLD; †—*p* < 0.05; ‡—*p* < 0.01; #—*p* < 0.001; ** absolute and relative frequencies and *p*-value for Chi-square test for contingency tables.

**Table 2:** Biochemical and redox status markers of the population studied.

Marker	CG N = 49	MASLD N = 50	MASLD + T2D N = 48	*p*
Glucose, mmol/L *	5.1 (4.8–5.4)	5.4 (4.9–5.9) a†	7.5 (6.0–10.2) a#, b#	<0.001
HbA1C, %	5.1 ± 0.34	5.4 ± 0.41	8.5 ± 2.32 a#, b#	<0.001
Total cholesterol, mmol/L	5.46 ± 1.33	5.31 ± 1.01	4.91 ± 1.22	0.081
TG, mmol/L *	0.90 (0.72–1.46)	1.30 (0.80–1.63)	1.88 (1.40–2.63) a#, b#	<0.001
HDL-cholesterol, mmol/L	1.68 ± 0.43	1.40 ± 0.27 a#	1.17 ± 0.29 a#, b†	<0.001
LDL-cholesterol, mmol/L	3.38 ± 1.14	3.28 ± 0.975	2.88 ± 1.05	0.081
Total bilirubin, µmol/L *	12.5 (9.3–17.7)	11.8 (7.4–17.1)	10.7 (8.5–14.7)	0.644
Direct bilirubin, µmol/L *	2.1 (1.6–2.7)	2.4 (1.8–4.0)	2.0 (1.5–2.5)	0.075
Total protein, g/L *	71 (68–75)	75 (72–77) a‡	68 (64–74) a‡, b#	<0.001
Albumin, g/L *	44 (43–46)	45 (44–49)	42 (39–44) a#, b#	<0.001
Uric acid, µmol/L *	268 (203–308)	373 (286–416) a#	308 (287–347) a#, b†	<0.001
Creatinine, µmol/L *	68 (58–85)	77 (63–87)	72 (59–80)	0.393
Urea, mmol/L *	5.0 (4.3–5.7)	5.2 (4.2–6.1)	5.1 (4.2–6.2)	0.789
ALT, U/L *	19 (16–25)	32 (19–51) a#	25 (17–47) a‡	<0.001
GGT, U/L *	15 (12–22)	36 (25–56) a#	30 (22–51) a#	<0.001
CRP, mg/L *	0.90 (0.40–2.25)	2.70 (1.35–5.30) a#	4.00 (2.00–6.90) a#	<0.001
TAS, µmol/L *	721 (695–745)	981 (950–1022) a#	870 (835–901) a#, b#	**<0.001**
TOS, µmol/L *	6.7 (3.2–8.3)	19.3 (14.6–25.5) a#	9.7 (5.8–13.3) a#, b#	**<0.001**
**O_2_^•−^,** **μ** mol/L NBT/min/L *	41 (33–47)	50 (46–56) a#	42 (36–49) b#	**<0.001**
IMA, ABSU *	0.347 (0.335–0.359)	0.473 (0.460–0.485) a#	0.467 (0.452–0.478) a#	**<0.001**

Abbreviations: CG, control group; T2D, type 2 diabetes; HbA1c, glycosylated hemoglobin; TG, triglycerides; HDL-cholesterol, high-density lipoprotein cholesterol; LDL-cholesterol, low-density lipoprotein cholesterol; ALT, alanine aminotransferase; AST, aspartate aminotransferase; ALP, alkaline phosphatase; GGT, γ-glutamyl transferase; CRP, C-reactive protein, TAS, total antioxidant status; TOS, total oxidant status; O_2_^•−^, superoxide anion radical; NBT, nitroblue tetrazolium; IMA, ischemia-modified albumin; ABSU, absorbance units. Data are presented as arithmetic mean ± SD; *p*-value for ANOVA; * median (interquartile range) and *p*-value for the Kruskal–Wallis; a—significant difference between MASLD and MASLD + T2D compared to the CG; b—significant difference between MASLD + T2D and MASLD; †—*p* < 0.05; ‡—*p* < 0.01; #—*p* < 0.001. Bold values are adjusted for age, sex, BMI, physical activity, antihypertensives and/or CVD therapy.

**Table 3:** Univariate binary logistic regression analysis of the examined markers’ association with the occurrence of MASLD.

Marker	OR	95% CI	Nagelkerke R^2^	*p*
Age, years	1.000	0.970–1.031	<0.001	0.995
BMI, kg/m^2^	1.392	1.196–1.620	0.345	<0.001
Waist circumference, cm	1.095	1.044–1.148	0.293	<0.001
Systolic blood pressure, mmHg	1.033	1.002–1.065	0.070	0.038
Diastolic blood pressure, mmHg	1.037	0.997–1.078	0.051	0.069
Glucose, mmol/L	2.611	1.182–5.766	0.091	0.018
HbA1C, %	7.290	1.779–29.871	0.151	0.006
Total cholesterol, mmol/L	0.899	0.626–1.293	0.005	0.567
TG, mmol/L	2.142	1.057–4.341	0.078	0.035
HDL-cholesterol, mmol/L	0.068	0.012–0.372	0.202	0.002
LDL-cholesterol, mmol/L	0.915	0.591–1.417	0.003	0.690
Total bilirubin, µmol/L	0.986	0.924–1.052	0.003	0.670
Direct bilirubin, µmol/L	1.369	0.947–1.979	0.049	0.094
Total protein, g/L	1.028	0.963–1.097	0.013	0.410
Albumin, g/L	0.994	0.975–1.013	0.011	0.558
Uric acid, µmol/L	1.014	1.007–1.021	0.302	<0.001
Creatinine, µmol/L	1.016	0.988–1.045	0.019	0.263
Urea, mmol/L	1.145	0.846–1.548	0.012	0.381
ALT, U/L	1.085	1.035–1.137	0.313	0.001
GGT, U/L	1.065	1.031–1.101	0.317	<0.001
CRP, mg/L	1.201	1.030–1.401	0.122	0.019
TAS, µmol/L	1.004	1.002–1.006	0.288	<0.001
TOS, µmol/L	1.127	1.020–1.245	0.158	0.019
O_2_^•−^, μmol/L NBT/min/L	1.014	0.997–1.031	0.098	0.040
IMA, ABSU	1.006	1.003–1.009	0.194	<0.001
miR-21 expression	8.337	1.707–40.722	0.126	0.009
miR-34a expression	7.512	1.495–37.742	0.149	0.014
miR-122 expression	1.849	0.970–3.526	0.070	0.062

Abbreviations: OR, odds ratio; CI, confidence interval; BMI, body mass index; HbA1c, glycosylated hemoglobin; TG, triglycerides; HDL-cholesterol, high-density lipoprotein cholesterol; LDL-cholesterol, low-density lipoprotein cholesterol; ALT, alanine aminotransferase; GGT, γ-glutamyl transferase; CRP, C-reactive protein; TAS, total antioxidant status; TOS, total oxidant status; NBT, nitroblue tetrazolium; O_2_^•−^, superoxide anion radical; IMA, ischemia-modified albumin; miR-, micro ribonucleic acid.

**Table 4:** Univariate binary logistic regression analysis of the examined markers’ association with the occurrence of T2D in MASLD.

Marker	OR	95% CI	Nagelkerke R^2^	*p*
Age, years	1.047	1.013–1.083	0.105	0.007
BMI, kg/m^2^	1.040	0.948–1.140	0.010	0.405
Waist circumference, cm	1.016	0.976–1.057	0.013	0.450
Systolic blood pressure, mmHg	0.997	0.971–1.024	0.001	0.835
Diastolic blood pressure, mmHg	1.000	0.970–1.031	0.000	0.991
Glucose, mmol/L	3.535	1.924–6.495	0.519	<0.001
HbA1C, %	13.187	3.242–53.635	0.728	<0.001
Total cholesterol, mmol/L	0.731	0.496–1.079	0.040	0.115
TG, mmol/L	2.409	1.325–4.381	0.164	0.004
HDL-cholesterol, mmol/L	0.063	0.010–0.386	0.179	0.003
LDL-cholesterol, mmol/L	0.673	0.419–1.083	0.051	0.103
Total bilirubin, µmol/L	1.001	0.953–1.052	0.000	0.960
Direct bilirubin, µmol/L	0.573	0.370–0.886	0.118	0.012
5Total protein, g/L	0.920	0.854–0.991	0.098	0.028
Albumin, g/L	0.624	0.500–0.780	0.470	<0.001
Uric acid, µmol/L	0.993	0.986–1.000	0.073	0.047
Creatinine, µmol/L	0.992	0.969–1.016	0.007	0.501
Urea, mmol/L	1.034	0.792–1.349	0.001	0.807
ALT, U/L	1.001	0.990–1.012	0.000	0.878
GGT, U/L	1.002	0.993–1.010	0.002	0.707
CRP, mg/L	1.054	0.968–1.149	0.032	0.226
TAS, µmol/L	0.998	0.996–1.000	0.066	0.033
TOS, µmol/L	0.982	0.949–1.016	0.038	0.300
O_2_^•−^, μmol/L NBT/min/L	0.994	0.982–1.007	0.012	0.360
IMA, ABSU	1.000	0.997–1.003	<0.001	0.939
miR-21 expression	0.036	0.005–0.256	0.218	0.001
miR-34a expression	0.932	0.463–1.877	0.001	0.844
miR-122 expression	0.995	0.702–1.411	<0.001	0.978

Abbreviations: OR, odds ratio; CI, confidence interval; BMI, body mass index; HbA1c, glycosylated hemoglobin; TG, triglycerides; HDL-cholesterol, high-density lipoprotein cholesterol; LDL-cholesterol, low-density lipoprotein cholesterol; ALT, alanine aminotransferase; GGT, γ-glutamyl transferase; CRP, C-reactive protein; TAS, total antioxidant status; TOS, total oxidant status; O_2_^•−^, superoxide anion radical; NBT, nitroblue tetrazolium; IMA, ischemia-modified albumin; miR-, micro ribonucleic acid.

**Table 5:** Factors extracted by PCA with percentage of variability and variables’ loadings.

Factors	Variables (Loadings)	Factor Variability (%)
Epigenetic liver-specific-related factor	miR-122 (0.877)	23.3
	miR-34a (0.846)	
	ALT (0.841)	
	GGT (0.625)	
	TG (0.531)	
Cardiometabolic antioxidant-related factor	HDL-cholesterol (−0.710)	14.4
	TAS (0.709)	
	BMI (0.615)	
	CRP (0.570)	
Redox-related factor	O_2_^•−^ (−0.711)	10.2
	TOS (0.561)	
	IMA (0.551)	
Age–epigenetic-related factor	Age (0.758)	10.1
	miR-21 (−0.743)	

Abbreviations: ALT, alanine aminotransferase; GGT, γ-glutamyl transferase; TG, triglycerides; HDL-cholesterol, high-density lipoprotein cholesterol; TAS, total antioxidant status; BMI, body mass index; CRP, C-reactive protein; O_2_^•−^, superoxide anion radical; TOS, total oxidant status; IMA, ischemia-modified albumin.

**Table 6:** Univariate binary logistic regression analysis of the association between PCA-derived factors and the occurrence of MASLD and T2D in MASLD.

**Predictors Towards MASLD**	**B**	**SE**	**Unadjusted** **OR (95%CI)**	***p***	**Nagelkerke** **R^2^**
Epigenetic liver-specific-related factor	1.071	0.481	2.918 (1.138–7.486)	0.026	0.165
Cardiometabolic antioxidant-related factor	1.524	0.432	4.592 (1.968–10.711)	<0.001	0.348
Redox-related factor	0.073	0.277	1.075 (0.625–1.850)	0.793	0.001
Age–epigenetic-related factor	−0.279	0.260	0.756 (0.454–1.259)	0.283	0.025
**Predictors towards T2D in MASLD**	**B**	**SE**	**Unadjusted** **OR (95%CI)**	***p***	**Nagelkerke** **R^2^**
Epigenetic liver-specific-related factor	0.060	0.227	1.062 (0.680–1.658)	0.793	0.002
Cardiometabolic antioxidant-related factor	0.374	0.315	1.454 (0.784–2.698)	0.235	0.034
Redox-related factor	0.163	0.239	1.178 (0.738–1.879)	0.493	0.011
Age–epigenetic-related factor	1.187	0.390	3.279 (1.527–7.040)	0.002	0.277

Abbreviations: B, regression coefficient; SE, standard error; OR, odds ratio; CI, confidence interval; T2D, type 2 diabetes.

## Data Availability

The data presented in this study are available on request from the corresponding author. The data are not publicly available due to privacy and ethical considerations.

## Supplementary Materials

The supporting information can be downloaded at: https://www.mdpi.com/article/10.3390/ijms26062392/s1.
