# Supplementary material for: Expression Analysis of Circulating miR-21, miR-34a and miR-122 and Redox Status Markers in Metabolic Dysfunction-Associated Steatotic Liver Disease Patients with and Without Type 2 Diabetes
Source: Int J Mol Sci. 2025 Mar 7;26(6):2392. doi: 10.3390/ijms26062392 (PMC11942408; doi:10.3390/ijms26062392)
Supplement: Supplementary file 1 [file ijms-26-02392-s001.zip › ijms-3453211-supplementary.pdf]

Table S1. Multivariate binary logistic regression analysis of the association between examined markers and MASLD occurrence.

Model 1	OR	95% CI	Nagelkerke R^2	P
BMI, kg/m^2	1.103	0.828 - 1.470	0.706	0.503
Hypertension and CVD	8.970	0.890 - 90.439		0.063
Glucose, mmol/L	7.306	1.249 - 42.719		0.027
TG, mmol/L	0.629	0.126 - 3.156		0.573
HDL-cholesterol, mmol/L	0.127	0.004 - 3.784		0.234
ALT, U/L	1.090	0.976 - 1.217		0.127
GGT, U/L	1.016	0.950 - 1.086		0.641
CRP, mg/L	0.989	0.761 - 1.287		0.937
TAS, µmol/L	1.005	1.001 - 1.009		0.012
TOS, µmol/L	0.997	0.946 - 1.051		0.908
IMA, ABSU	51.025	0.073 - 35732.089		0.239
miR-21 expression	0.658	0.035 - 12.559		0.781
**Model 2**	**OR**	**95% CI**	**Nagelkerke R^2**	**P**
BMI, kg/m^2	1.138	0.830 - 1.562	0.723	0.422
Hypertension and CVD	3.505	0.265 - 46.384		0.341
Glucose, mmol/L	4.169	0.605 - 28.717		0.147
TG, mmol/L	0.380	0.060 - 2.393		0.303
HDL-cholesterol, mmol/L	0.110	0.003 - 3.563		0.214
ALT, U/L	1.121	0.973 - 1.292		0.114
GGT, U/L	1.054	0.971 - 1.143		0.208
CRP, mg/L	0.887	0.643 - 1.223		0.464
TAS, µmol/L	1.004	1.000 - 1.008		0.051
TOS, µmol/L	1.008	0.947 - 1.074		0.792
IMA, ABSU	179.394	0.153 - 210453.024		0.150
miR-34a expression	0.381	0.015 - 9.950		0.562

Abbreviations: OR, odds ratio; CI, confidence interval; BMI, body mass index; CVD, cardiovascular disease; TG, triglycerides; HDL-cholesterol, high-density lipoprotein cholesterol; ALT, alanine aminotransferase; GGT, γ-glutamyl transferase; CRP, C-reactive protein; TAS, total antioxidant status; TOS, total oxidant status; IMA, ischemia-modified albumin; ABSU, absorbance units, miR-, micro ribonucleic acid.

Table S2. Multivariate binary logistic regression analysis of the association between examined markers and the occurrence of T2D in MASLD.

Model 3	OR	95% CI	Nagelkerke R^2	P
Age, years	1.041	0.989 - 1.095	0.428	0.121
TG, mmol/L	1.823	0.900 - 3.692		0.096
HDL-cholesterol, mmol/L	0.078	0.009 - 0.671		0.020
TAS, µmol/L	0.998	0.996 - 1.001		0.148
miR-21 expression	0.050	0.004 - 0.668		0.023

Abbreviations: OR, odds ratio; CI, confidence interval; TG, triglycerides; HDL-cholesterol, high-density lipoprotein cholesterol; TAS, total antioxidant status; miR-, micro ribonucleic acid.

Table S3. Univariate binary logistic regression analysis of the association between factors and MASLD occurrence.

Factor	OR	95% CI	Nagelkerke R^2	P
Epigenetic-liver specific-related factor	2.915	1.137-7.471	0.165	0.026
Cardiometabolic antioxidant-related factor	4.604	1.972-10.753	0.349	<0.001
HSI	1.288	1.149-1.444	0.376	<0.001
FLI	1.052	1.026-1.079	0.394	<0.001

Abbreviations: OR, odds ratio; CI, confidence interval; hepatic steatosis index (HSI); fatty liver index (FLI).
